# Control freaks—signals and cues governing the regulation of virulence in attaching and effacing pathogens

**DOI:** 10.1042/BST20180546

**Published:** 2018-12-17

**Authors:** Natasha C.A. Turner, James P.R. Connolly, Andrew J. Roe

**Affiliations:** Institute of Infection, Immunity and Inflammation, University of Glasgow, Glasgow G12 8TA, U.K.

**Keywords:** EHEC, gene expression and regulation, T3SS, virulence

## Abstract

Enterohaemorrhagic *Escherichia coli* (EHEC) mediates disease using a type 3 secretion system (T3SS), which is encoded on the locus of enterocyte effacement (LEE) and is tightly controlled by master regulators. This system is further modulated by a number of signals that help to fine-tune virulence, including metabolic, environmental and chemical signals. Since the LEE and its master regulator, Ler, were established, there have been numerous scientific advancements in understanding the regulation and expression of virulence factors in EHEC. This review will discuss the recent advancements in this field since our previous review, with a focus on the transcriptional regulation of the LEE.

## Introduction

*Escherichia coli* is a Gram-negative bacterium, highly adapted to survive within the gastrointestinal tract of multiple hosts [1]. Several pathotypes are classed as enteric diarrheal pathogens, such as Enterohaemorrhagic *E. coli* (EHEC) and most notably the O157:H7 serotype. EHEC is a subset of Shiga-toxin *E. coli* (STEC) that can cause diarrheal infections and in severe cases lead to haemolytic uraemic syndrome (HUS). These infections often affect the western world, whereas a closely related strain, enteropathogenic *E. coli* (EPEC), affects primarily children under 2 years old in developing countries [2,3]. HUS develops from the production of Shiga toxins (Stx) from EHEC, which are expressed in the intestine [4]. Cattle are the natural reservoir for EHEC infections, which they colonise asymptomatically, and previously outbreaks have been associated with contaminated meat; however, EHEC can also be contracted from other animals as well as fresh plant produce [5–9].

EHEC and EPEC infections are defined by the formation of characteristic attaching and effacing (AE) lesions on the surface of the host epithelia, which are dependent on the T3SS [10,11]. The genes encoding this T3SS are carried on the locus of enterocyte effacement (LEE) pathogenicity island (PAI) which consists of 41 open reading frames spread across five polycistronic operons, *LEE1 to LEE5*, which are under the control of the LEE-encoded master regulator (Ler), encoded on *LEE1* and regulated by numerous transcription factors in response to environmental stimuli [12,13]. Protein secretion is crucial for the virulence of EHEC, enabled using the LEE-encoded T3SS. This process results in translocation of several effector proteins from the bacterial cytosol into the host cell where they subvert normal function and facilitate close attachment of the bacteria [14]. The natural murine pathogen *Citrobacter rodentium* similarly relies on the LEE-encoded T3SS for host colonisation and has been adopted as the relevant surrogate animal model to study EHEC infections *in vivo* [15].

EHEC virulence factors are tightly regulated and controlled by numerous other regulators and systems designed to sense the environment. There have been extensive reviews published surrounding regulation of EHEC virulence factors in the last 20 years [2,16,17]. This review will discuss the most recent scientific advancements in the field of LEE regulation in response to environmental factors.

## Regulation of virulence through chemical and nutritional signals in the intestine

There has been much interest surrounding microbiota signalling and the host metabolites that can affect the virulence of pathogenic *E. coli* [18–21]. EHEC is exposed to a large number of chemical and nutritional signals within the gastrointestinal tract, sensing its environment and regulating gene expression based upon those factors. Regulation of key virulence factors such as the expression of the T3SS is often reliant on these small molecule signals, which help to dictate the appropriate location to initiate attachment. This allows the pathogen to carve a niche within an extremely diverse environment and establish a successful colony.

These signals can often be produced from the microbiota itself. Indeed, in relation to EHEC, it has been shown that the gut commensal *Bacteroides thetaiotaomicron* can enhance virulence gene expression in EHEC through the sugar sensitive transcription factor Cra [22]. *B. thetaiotaomicron* catabolises larger sugar molecules which creates a more gluconeogenic environment, which through Cra, initiates virulence gene expression [22]. It has also been shown that the animal mouse model pathogen *C. rodentium* is reliant on gut microbiome commensals for colonisation of the colonic mucosa [23]. Specific dysbiosis of the microbiota displaces *C. rodentium,* showing that *C. rodentium* relies on commensals to colonise specific physiological regions [23]. Furthermore, as a part of the broader microbiome, bacteriophages can also affect the virulence of EHEC. While lysogenic, it has been shown that there is a decrease in T3SS expression and the Stx2 prophage can repress the T3SS by restricting Ler-mediated activation involving the CII regulator [24]. Lysogenic bacteriophages also produce Cro, a transcription factor which can activate the T3SS in EHEC [25]. This highlights the complex system of how the microbiome itself can affect EHEC virulence gene regulation and potentially can be exploited as a treatment option.

Quorum sensing is important for virulence for bacterial pathogens as they use bacterially produced chemical signals, as well as those produced by the host, to sense the environment and co-ordinate gene expression [26]. The eukaryotic hormones epinephrine and norepinephrine are perceived through quorum sensing, regulating virulence factors in EHEC [27,28]. Sensing of epinephrine and norepinephrine is achieved through two histidine kinase response regulator systems, QseBC and QseEF, which in turn activate QseA, a Lys-R type regulator that activates transcription of *ler,* by binding to the P1 and P2 promoter region [29–32]. QseC is a global regulator in EHEC and can directly sense and autophosphorylate in response to epinephrine and norepinephrine [33]. QseC triggers a signalling cascade phosphorylating three response regulators; its cognate response regulator QseB, and two non-cognate response regulators QseF and KdpE, which in turn can act upon *ler* and motility functions [30,31,33–35]. Recent research into the periplasmic sensing domain of QseC has suggested that modifications in this domain can affect its ability to phosphotransfer to its response regulator, furthermore, hypothesising that QseC has a different affinity for each of its response regulators [27]. The second histidine kinase response regulator that can sense epinephrine and norepinephrine, along with phosphate and Sulfate signals from the environment is QseEF. It has been found that an outer membrane lipoprotein, *qseG*, is co-transcribed with *qseEF* [31]. QseG is required for full virulence in the *C. rodentium* mouse model and interacts with proteins in relation to the T3SS [28]. Interestingly, it appears that QseG modulates the expression of the QseEF two-component system, with the *ΔqseG* mutant increasing the expression of *qseE* [28]. Both QseC and QseE are required for LEE activation with the respective mutants attenuated in a *C. rodentium* mouse model, suggesting how vital for virulence the quorum sensing of host signals is during an infection [36].

Mucin-derived sugars are a useful carbon source for some bacteria, allowing them to exploit particular niches. *E. coli* can utilise many of these carbon sources including *N*-acetyl-galactosamine, which serve dual roles as chemical signals in EHEC that can affect virulence gene regulation. It has been shown that *N*-acetylglucosamine (NAG) and *N*-acetylneuraminic acid (NANA) inhibit EHEC colonisation through the activity of NagC, an activator of the LEE1 operon [37,38]. When NAG and NANA are catabolised, NAG-6 phosphate is produced, NagC is an NAG-6 phosphate sensing protein [38,39]. Mutations in *nagC* showed a decreased expression of LEE genes, potentially due to NagC being able to alter the expression of *ler* [38]. This has also been shown *in vivo* where NagC promotes EHEC colonisation [38]. This is an example of how nutrient availability can affect the virulence gene regulation of EHEC. A further dietary signal that has been recently shown to influence EHEC virulence gene regulation is iron [40]. Using the *C. rodentium* mouse model, it was shown that dietary iron influenced physiological changes in the host, which led to the attenuation of virulence and suppressed virulence gene expression [40]. When iron was added to the diet, it conferred complete protection against the *C. rodentium* infection with no development of colonic pathology and inflammation that occurs during an infection [40]. It was, however, found that the *C. rodentium* were still present but asymptomatic, due to down-regulation of virulence factors [40]. This was due to a mechanism involving the dietary iron increasing glucose availability and therefore reducing glucose absorption through inducing insulin resistance, which suppressed the expression of the LEE [40]. This work highlights how critical anti-virulence factors are, as well as showing that host metabolism and virulence gene expression are inherently interlinked.

Members of the Enterobacteriaceae often utilise microbiota-derived nutrients as energy sources and signals for virulence regulation in parallel during infection of the intestinal niche [41]. A recent study probed the transcriptome of *C. rodentium* during infection of its natural murine host and identified a metabolic signature that was specific to this niche. Metabolism of the microbiota-derived fermentation by-product 1,2-propanediol was highly induced during murine infection. *C. rodentium* encodes the genes required for catabolism of 1,2-propanediol and accordingly can utilise it as a source of energy. Furthermore, the study revealed that 1,2-propanediol increased expression of the LEE T3SS as well as a suite of non-LEE-encoded effectors. Importantly, this regulation was found to be entirely dependent on the active metabolism of 1,2-propanediol, which results in the endogenous production of the short chain fatty acid propionate that acts as the signal for LEE expression. Finally, the study demonstrated that 1,2-propanediol metabolism was required by *C. rodentium* for maximal colonisation *in vivo* and that this was governed entirely by its effects on T3SS regulation and not as a result of decreased fitness due to the inability to utilise this nutrient source. The present study is a prime example of host-specific factors that are critical for fine-tuning virulence and therefore promote persistence within the host-niche [42].

The interactions between metabolism and virulence have dramatic effects on how EHEC has evolved to colonise its preferred niche. This has been shown specifically with the host metabolite d-serine [43]. d-serine is found in various parts of the human body, with a range of high concentrations found in the urinary tract from 3 to 115 µg/ml [44] but low concentrations in the gut. d-serine can be used as a carbon source and affects gene expression in certain strains of *E. coli*, for example, urinary pathogenic *E. coli* (UPEC). However, d-serine cannot be metabolised by EHEC, is bacteriostatic and has been shown to down-regulate LEE expression through a global shift in gene expression that affects multiple transcription factors [43]. While the precise mechanism of d-serine sensing is unclear, it was also discovered that EHEC expresses a functional inner membrane amino acid transporter with an affinity for d-serine. This transporter is also partially regulated by a Lys-R type transcriptional regulator, YhaJ, which has also been adapted to promote LEE expression [45]. This system demonstrates the versatility of the *E. coli* genome in regulating pathotype specific process but also illustrates how host metabolites, such as amino acids affect niche specificity through regulating colonisation. More recently, it has been shown that a cysteine-responsive regulator CutR, interacts with YhaO to increase LEE gene activation and expression [46]. CutR also interacts indirectly with FadR, a repressor of the LEE, by activating the expression of *fadL* which encodes a transporter for fatty acids which inhibit FadR [46]. CutR and FadR are transcription factors that regulate metabolism in EHEC and are novel regulators of the EHEC virulence status [46]. These novel pathways highlight how important a wide variety of signals are for virulence gene expression within EHEC and how they intersect with each other to co-ordinate virulence.

## Cellular regulation that affects upon the LEE

As well as specific environmental sensing systems, regulation of the LEE overlaps with transcriptional networks under the influence of global regulators not encoded within the island itself. For instance, the histone-like nucleoid structuring protein H-NS is a global regulator involved in silencing the transcription of foreign DNA [47]. H-NS has been shown to regulate 983 genes in EHEC and represses the LEE under non-inducing conditions [48]. Ler, the first gene encoded on *LEE1*, is a paralog of H-NS and can relieve H-NS repression of the T3SS. Recently, the mechanism underlying Ler alleviation of H-NS repression has been elucidated, with Ler binding upstream of the *LEE5* promoter, displacing the pre-bound H-NS, suggesting that H-NS has a lower affinity to the *LEE5* promoter [49].

Pch, a small transcriptional regulator, has also been shown to regulate the LEE [50,51]. Recently, the mechanism has clarified that Pch can activate the *LEE1* promoter through the reduced binding of H-NS at the *LEE1* promoter site [52]. Pch is required for the full activation of LEE genes and can activate the *LEE1* promoter by disrupting silencing by the global regulators H-NS, StpA, Hha and YdgT [52]. Pch also enhances Ler-mediated transcription; however, the mechanism for this is unknown [52]. Another factor that can regulate virulence of the LEE is Mpc (multiple point controller). Mpc is encoded as the first gene on the *LEE3* and can repress the activity of *ler* when overexpressed [53,54].

GrvA (global regulator of virulence A), a Tox-R family transcriptional regulator, is also involved in the regulation of the LEE. GrvA works in conjunction with RcsB, a regulator which has been shown to activate and repress the LEE; however, until recently it has not been understood how this mechanism works [55,56]. *grvA* is directly activated by RcsB, with RcsB hypothesised to bind at *grvA* promoter region, although this is temperature sensitive [56]. GrvA positively regulates the LEE by indirectly down-regulating GadE, a regulator of acid tolerance and known repressor of *ler* [56,57]. GrvA repression of *gadE* was dependent on an intact *gadW* [56].

Within EHEC, there is also a secondary non-functional *E. coli* type three secretion system 2 (ETT2), which has a putative regulator, EtrB [58]. EtrB has been shown to directly interact with *ler* to activate LEE expression and is positively regulated by QseA [58]. ETT2 contains five predicted transcription factors including EtrA and EivF which have been shown to repress LEE expression [59]. EtrB modulates the expression of the T3SS by repressing expression of *etrA* and *eivF* and positively interacting directly with the LEE1 promoter region [58].

## Regulation of the T3SS by mechanosensation

As well as sensing nutrients or small molecules, a recent concept has emerged in the field of LEE regulation that focuses on sensing of physical cues, known as ‘mechanosensing’ [60,61]. Initial direct attachment to host cells generates a mechanical cue that, by means of the regulator protein GrlA, is required to fully activate the LEE [60]. GrlA, the global regulator of *ler* also encoded on the LEE, is an MerR-like transcription factor and is repressed by GrlR [62,63]. GrlA activates LEE expression through interactions with *ler* and is repressed by GlrR, which forms a tight complex with GrlA blocking it from binding to *ler* promoter region [64]. It has been shown that free GrlA cannot fully activate LEE expression and further mechanosensing cues are needed for full activation, such as host cell attachment and fluid shear [60]. This highlights that along with environmental and metabolic signals, physical signals are also used in the gene regulation of virulence factors in EHEC.

Physical cues are also critical for the expression of effector proteins in EHEC infection. NleA is a non-LEE-encoded effector protein secreted by the T3SS, contributing to host colonisation and modulation [65]. Katsowich et al. [66] have recently shown in EPEC that NleA is not expressed until the bacterium is in contact with the host cell. This is done post-transcriptionally by the T3SS chaperone CesT, which antagonises CsrA. CsrA inhibits translation of NleA by binding to a CsrA recognition sequence on the *nleA* 5′UTR [66]. CesT binds to CsrA upon contact with the host cell, resulting in NleA expression [66]. This emphasises the recent research being done on physical cues for bacteria and how they can affect gene regulation of virulence factors. Furthermore, the LEE is subject to extensive post-transcriptional and post-translational regulation. However, this has very recently been reviewed in detail and will not be discussed further here [67].

## Environmental stress and pressures affecting gene regulation

When EHEC enters the host, it encounters multiple environmental stresses that can affect gene regulation, including pH and oxidative stress. Within the stomach, the environment is acidic, with pH ranges often between 1.5 and 2.5 [68]. To survive this, EHEC has evolved at least three acid resistance systems, including the glutamate-decarboxylase system (GAD) [69]. This system is controlled by the Gad genes and is the most effective acid resistance system in *E. coli* [70]. GrvA has recently been shown to be a novel repressor of this system, with a *grvA* deletion up-regulating the Gad regulatory genes, *gadE*, *gadW* and *gadX*, increasing acid survival by the GAD system [56].

A wide range of environmental stimuli including pH changes and alterations in the membrane can affect the Cpx envelope stress response of EHEC [71,72]. The Cpx response is controlled by a two-component system, CpxA the histidine kinase and CpxR the response regulator. This system has been found to regulate LEE expression in EHEC, as it was found that high levels of CpxR, in the absence of CpxA, repressed transcription of *grlA* and *ler* [73]. CpxR activates the expression of *rpoH*, which is known to regulate virulence and regulates the *lon* protease gene [73,74]. It was found that even though an increase in CpxR did not appear to increase transcription of *lon*, Lon was involved in negatively regulating the LEE by down-regulating *ler*, *grlA* and *nleA* genes [73]. In contrast, however, it has been reported that the expression of NlpE *in vitro* activates the Cpx response, with LEE expression up-regulated via LrhA, through the up-regulation of *pchA* and *pchB* [75,76]. This system potentially shows how several environmental cues can converge on one pathway which can affect virulence gene regulation in EHEC.

Oxygen concentration is a key indicator for EHEC to determine its environment, as its availability is sparse in the gut adding to the competitive pressure against commensal anaerobes. Furthermore, oxygen can act as a cue influencing virulence gene regulation, including the LEE [77,78]. When oxygen is absent, LEE gene expression is repressed through KdpE and FusR [77]. Cra, a global regulator, directly binds to *ler* when oxygen is available activating the expression of all LEE genes, with this activation possibly enhanced by interaction with KdpE [77]. This is key in the tight regulation of virulence factors only activating when environmental conditions are optimal. Furthermore, these transcription factors are sugar sensing and therefore can also regulate the expression of the T3SS from sensing different mucin-derived sugars [77]. It was shown that the expression of EspB, an LEE-encoded translocated effector, was negatively affected when the sole carbon sources were galactose, fructose and fucose, for example, in comparison with when pyruvate and sialic acid were the sole carbon sources, where an increase in EspB secretion was observed [77]. These sugars are produced in different parts of the gut and therefore detecting the differences in mucin-derived sugars allows EHEC to monitor nutrient availability and express the T3SS in ideal conditions [77].

Oxygen can also be regarded as a requirement for certain steps in central metabolism, such as the activity of the succinate dehydrogenase complex, which converts succinate to fumarate. Indeed, it has been recently demonstrated that *Salmonella* Typhimurium takes advantage of infection-induced oxygenation of the mucosa to complete the TCA cycle *in vivo* [79]*.* Using a multi-omics analysis, it was demonstrated that a disruption in the succinate dehydrogenase complex (Sdh) showed significantly attenuated pathogenicity of EHEC in a *Caenorhabditis elegans* model, with *sdhA* required for full toxicity [80]. The Sdh complex converts succinate to fumarate in the TCA cycle, and when fumarate was supplemented to the *sdhA* mutant, the attenuation was reversed [80].

A recent study has further highlighted how vital oxygen availability is for pathogenicity. *C. rodentium* subverts carbon metabolism mechanism in intestinal epithelial cells, allowing for a greater concentration of oxygen at the apical surface, creating a favourable environment for *C. rodentium* colonisation [81]. It was demonstrated that *C. rodentium* disrupted the TCA cycle by inhibiting the supply of substrates and caused mitochondrial ATP production to stop [81]. These disruptions led to increased oxygen concentrations at the mucosal surface, which is a key signal for expression of the T3SS [77,81]. A further study showed that *C. rodentium* also increased oxygenation at the mucosal surface by triggering colonic crypt hyperplasia, a pathological hallmark of the infection, through the T3SS [82]. This colonic crypt hyperplasia, induced by the T3SS, increased the growth of *C. rodentium*, creating a favourable niche for expansion [82]. Furthermore, transcriptional analyses recently reported using *C. rodentium in vivo* revealed significant up-regulation of oxygen-dependent metabolic genes (such as *sdh*, as described above) during infection [42]. Given that these two mechanisms have been recently described for increased oxygenation at the mucosal surface, this suggests how vital oxygen is as an environmental signal, regulating expression of virulence factors and promoting a competitive advantage to A/E pathogens.

As well as acid and oxidative changes EHEC have to overcome the immune system. The immune system can be triggered by many stimulants, including lipid A, through which the modulation of can be used to elude the immune response [83,84]. Along with regulating the T3SS, Pch and Ler can regulate lipid A modification [85]. Lipid A modification can be due to dephosphorylation and acylation and are controlled by enzymes such as LpxR [85–87]. *lpxR* was shown to decrease phagocytosis of EHEC through 3′-O-deacylase activity [85]. LpxR is transcribed along with LEE genes and requires both Pch and Ler for activation [85]. OmpT is also important in defence against the immune system, protecting EHEC from host produced anti-microbial peptides, and also appears to be regulated by Pch and Ler [88].

## Epigenetic regulation of the LEE

Rapid response regulation is key for bacteria to respond to environmental stresses through modulation of gene expression, with recent work highlighting how these responses can regulate the expression of virulence factors. Work completed in EPEC has suggested that there is a ‘bet-hedging’ strategy where proteins are regulating the bimodal expression of the LEE and virulence in general [89,90]. A hysteretic memory switch, controlled in part by the *per* operon, was shown to regulate bimodality of virulence expression, with the coexistence of non-virulent and hyper-virulent subpopulations [89]. This has potentially created a ‘bet-hedging’ strategy in which the two phenotypes are stable under non-activating conditions [89]. Furthermore, Leh et al. [90] have shown that bacterial-chromatin structural proteins, H-NS and Ler can regulate the bimodal expression of the LEE. This again allows for two subpopulations with high and low states of expression [90]. These studies highlight how epigenetic regulation can affect and regulate bacterial virulence with respect to A/E pathogens.

## Final remarks

In recent years, a wealth of knowledge has been revealed regarding the mechanisms of virulence gene regulation in EHEC and associated pathogens. This has contributed greatly to our understanding of LEE regulation and the pathogenicity of EHEC. This has been further enhanced by the use of the *C. rodentium* mouse model to study host-specific regulation and responses, as well as the emergence of zebrafish as a vertebrate model host [91]. These models, along with a continued effort to decipher details molecular mechanisms of virulence gene regulation are revealing the complex interplay between the host, the microbiome and the pathogen during infection. This knowledge will hopefully contribute to the design of a new treatment against EHEC. This is of particular importance given that the use of antibiotics to treat EHEC infection is inadvisable. Future work is underway exploring the concept of anti-virulence as a therapy, which often relies on a detailed understanding of virulence gene regulatory networks and offers a promising alternative to traditional antibiotics [92,93].

**Figure 1.**
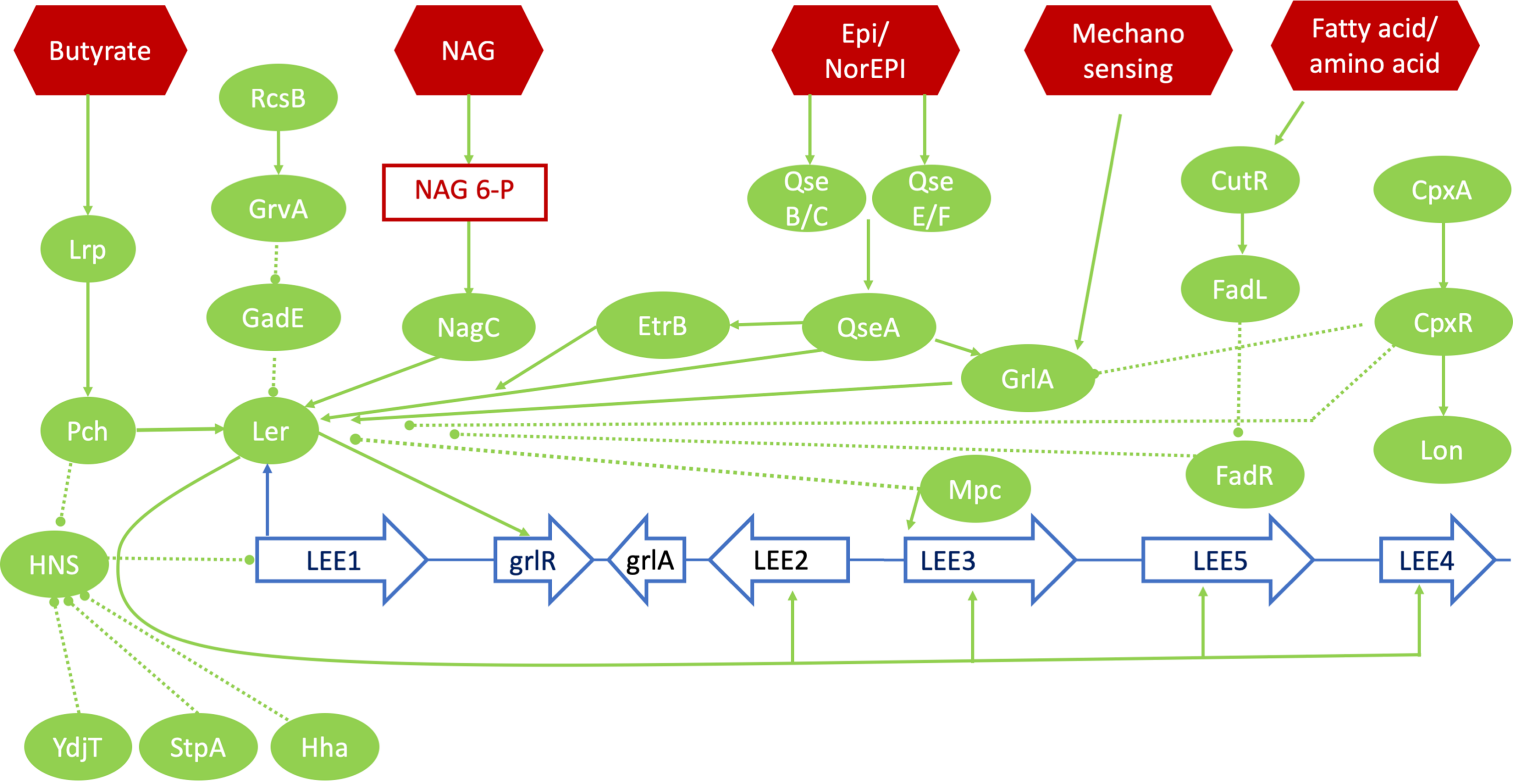
Overview of regulatory pathways discussed in this review. Key environmental signals that act as inputs are coloured red. Proteins are coloured green with connections indicated by arrows; solid for positive regulators and dashed lines for negative regulators. The five polycistronic operons of the LEE are coloured blue. Abbreviations: NAG/NAG 6-P: *N*-acetylglucosamine and *N*-acetylglucosamine 6 phosphate; Epi/NorEpi: epinephrine and norepinephrine.

## Abbreviations

AE: attaching and effacing
EHEC: Enterohaemorrhagic *Escherichia coli*
EPEC: enteropathogenic *E. coli*
GAD: glutamate-decarboxylase
HUS: haemolytic uraemic syndrome
LEE: locus of enterocyte effacement
NAG: N-acetylglucosamine
NANA: N-acetylneuraminic acid
PAI: pathogenicity island
STEC: Shiga-toxin *E. coli*
T3SS: type 3 secretion system
UPEC: urinary pathogenic *E. coli*
